# 人源化靶向CD19 CAR-T细胞治疗复发/难治急性B淋巴细胞白血病的有效性及安全性

**DOI:** 10.3760/cma.j.issn.0253-2727.2022.08.006

**Published:** 2022-08

**Authors:** 凤美 宋, 永仙 胡, 明明 张, 文俊 吴, 惠君 徐, 鸿声 张, 河 黄, 国庆 魏

**Affiliations:** 1 浙江大学医学院附属第一医院骨髓移植中心，浙江大学良渚实验室，浙江大学血液学研究所，浙江省干细胞与细胞免疫治疗工程实验室，浙江 311100 Bone Marrow Transplantation Center, Institute of Hematology, The First Affiliated Hospital, College of Medicine, Zhejiang University, Zhejiang Province Engineering Laboratory for Stem Cell and Immunity Therapy, Zhejiang Laboratory for Systems & Precision Medicine, Zhejiang University Medical Center, Hangzhou 311100, China; 2 同济大学医学院附属肺科医院临床转化中心，上海 200438 Clinical Transformation Center, Shanghai Pulmonary Hospital, Tongji University School of Medicine, Shanghai 200438, China

**Keywords:** 嵌合抗原受体, 人源化, 急性淋巴细胞白血病, 难治, 复发, Chimeric antigen receptor T cell, Humanized, Acute lymphoblastic leukemia acute, Relapsed, Refractory

## Abstract

**目的:**

观察人源化靶向CD19嵌合抗原受体T细胞（CAR-T）治疗复发/难治急性B淋巴细胞白血病（R/R B-ALL）患者的有效性及安全性。

**方法:**

分析2020年2月至2021年7月于浙江大学医学院附属第一医院接受人源化靶向CD19 CAR-T细胞治疗的41例R/R B-ALL患者的有效性和安全性。

**结果:**

中位第15（9～47）天，41例患者的完全缓解率为95.1％（39/41），其中38例患者骨髓经流式细胞术检测微小残留病灶阴性。39例完全缓解的患者中17例未接受进一步治疗，70.6％（12/17）的患者在随访结束时仍处于缓解状态，输注最早的两例患者无进展生存期达12.6个月；另外17例患者缓解后行巩固性造血干细胞移植（10例）或CD22 CAR-T细胞序贯治疗（7例），76.5％（13/17）的患者在随访结束时仍处于缓解状态；其余5例患者未接受巩固性治疗，在CAR-T细胞治疗后中位第72（55～115）天复发。1年总生存率为73.6％（95％*CI* 55.2％～92.3％），1年无进展生存率为56.2％（95％*CI* 38.1％～75.2％）。所有患者均发生了细胞因子释放综合征，63.4％（26/41）为1～2级。3例患者发生免疫效应细胞相关神经毒性综合征。

**结论:**

人源化靶向CD19 CAR-T细胞能有效诱导R/R B-ALL患者获得完全缓解，且不良反应可耐受。

## Abstract (en)

**Objective:**

This study aimed to evaluate the safety and efficacy of humanized CD19-targeted chimeric antigen receptor T-cell（CAR-T）in patients with relapsed/refractory acute B cell lymphoblastic leukemia（R/R B-ALL）.

**Methods:**

The clinical data of 41 patients with R/R B-ALL treated with humanized CD19-targeted CAR-T cells in the First Affiliated Hospital of Zhejiang University School of Medicine from February 2020 to July 2021 were analyzed.

**Results:**

Cytokine release syndrome occurred in all patients, and 63.4％（26/41）were grades 1–2. Immune effector cell-associated neurotoxicity syndrome developed in three patients. On median day 15（9–47）, the complete remission rate was 95.1％（39/41）, of which 38 patients tested negative for bone marrow minimal residual disease detected by flow cytometry. Among the 39 patients with complete remission, 17 patients did not receive further treatment, and 70.6％（12/17）remained in remission at the end of follow-up, with a progression-free survival of 11.6 months of the two patients with the earliest infusion. Another 17 patients underwent consolidation allogeneic hematopoietic stem cell transplantation（10 cases）or CD22 CAR-T cell sequential therapy（seven cases）after remission, and 76.5％（13/17）of the patients were still in remission at the end of follow-up. The remaining five patients who did not receive consolidation therapy relapsed at a median of 72（55–115）days after CAR-T cell therapy.

**Conclusion:**

In patients with R/R B-ALL, the humanized CD19-targeted CAR-T cells had a high response and manageable toxicity.

急性淋巴细胞白血病传统治疗方法有化疗、造血干细胞移植、靶向药物治疗等，但这些治疗方案未能显著改善复发难治患者的预后[1]–[4]。近年来，嵌合抗原受体T细胞（CAR-T细胞）疗法的出现，为复发/难治患者提供了新的治疗策略。多项临床研究显示，靶向CD19 CAR-T细胞治疗复发/难治急性B淋巴细胞白血病（B-ALL）患者完全缓解率可达70％～90％[5]–[7]。然而较高的复发率和严重的细胞因子释放综合征（CRS）仍然是CAR-T细胞治疗过程中亟待解决的两大挑战。研究表明，鼠源化CAR结构中的单链可变片段中的抗原表位可以引起HLA限制性T细胞介导的免疫反应[8]。而通过改变CAR的框架或非互补决定区域使用人源化抗体片段可减低其免疫原性，从而使CAR-T细胞治疗过程中细胞因子的释放减少并且抗肿瘤活性增强[9]。本研究中，我们将我中心应用人源化靶向CD19嵌合抗原受体T细胞（hCART19s细胞）治疗41例复发/难治B-ALL患者的有效性及安全性报道如下。

## 病例与方法

1. 病例资料：纳入2020年2月至2021年7月于浙江大学医学院附属第一医院行hCART19s治疗的复发/难治B-ALL患者41例。患者的诊断依据WHO2016年分类标准。难治定义为诱导治疗结束未能取得完全缓解（CR）；复发定义为已取得CR的患者外周血或骨髓又出现原始细胞（比例>5％），或出现髓外疾病。本研究经浙江大学医学院附属第一医院伦理委员会批准（批件号：IIT20200058C），所有患者均知情同意并签署知情同意书。

2. CAR-T细胞制备及输注：本研究所用的CAR-T细胞为二代CAR-T细胞，采用人源化抗体序列，共刺激信号元件为4-1BB。制备的基本过程如下：①输注前10～14 d采集供者（4例既往接受过异基因造血干细胞移植的患者细胞来源为供者）或患者外周血T淋巴细胞；②在体外通过慢病毒载体将CAR结构片段转染进入T细胞，CD19 CAR中位转染效率为56.4％（8.2％～89.7％）；③进行体外分离、纯化和扩增。在CAR-T细胞输注前给予FC（氟达拉滨+环磷酰胺）方案预处理清除淋巴细胞，输注时间根据预处理前外周血白细胞数进行调整（若外周血WBC>10×10^9^/L，氟达拉滨30 mg·m^−2^·d^−1^、−5～−3 d，环磷酰胺500 mg·m^−2^·d^−1^、−2～−1 d；若外周血WBC≤10×10^9^/L，氟达拉滨30 mg·m^−2^·d^−1^、−3～−1 d，环磷酰胺500 mg·m^−2^·d^−1^、−2～−1 d）。hCART19s细胞的中位输注量为2.33（0.83～3.64）×10^6^/kg。

3. 疗效及不良反应评估：在患者输注CAR-T细胞后外周血中性粒细胞绝对计数>0.5×10^9^/L时进行评估，中位评估时间为15（9～47）d。根据NCCN指南疗效评估标准进行近期疗效评估。不良反应评估标准：CRS诊断及分级参照Lee等[10]的分级标准，免疫效应细胞相关神经毒性综合征（ICANS）分级参照ASBMT NT Consenus Grading分级标准，其他临床常见不良反应参照美国常见不良事件评价标准（CTCAE）5.0版。

4. 随访：通过门诊或住院复查、电话随访、检索病历的方式进行随访。随访时间截至2021年9月1日，中位随访时间为177（15～521）d。总生存（OS）期为CAR-T细胞输注至任何原因死亡或随访结束。无进展生存（PFS）期为CAR-T细胞输注后至疾病复发、死亡或随访终点。

5. 统计学处理：应用R 4.0.3版软件进行统计学分析。Kaplan-Meier生存分析计算1年OS及PFS率并绘制生存曲线。采用Spearman等级相关分析计算CRS等级与外周血指标峰值的相关性，0～2级与3～5级CRS组间差异比较采用Willcoxon法。双侧*P*<0.05为差异有统计学意义。

## 结果

1. 一般临床特征：共纳入41例复发/难治B-ALL患者，其中男24例，女17例，中位年龄44（13～74）岁。Ph^+^ALL 13例，既往均使用过酪氨酸激酶抑制剂治疗，其中10例伴T315I突变。9例患者有髓外病变，涉及中枢、睾丸、骨骼、软组织累及。CAR-T细胞输注前中位化疗5（1～16）次。6例患者既往接受过造血干细胞移植，1例为自体移植，其余5例为异基因移植。接受异基因移植的5例患者中除1例患者因供者无法再次行干细胞采集CAR-T细胞来源于自体外，其余4例患者CAR-T细胞来源均为供者。CAR-T细胞输注前患者的骨髓原始细胞比例中位数为59％（0～94％）。详见表1。

**表1:** 41例复发难治性急性B淋巴细胞白血病患者的临床特征

临床特征	例数	构成比（％）
性别		
男	24	58.5
女	17	41.5
年龄		
<30岁	13	31.7
≥30岁	28	68.3
CAR-T输注前骨髓肿瘤负荷		
<20％	13	31.7
≥20％	28	68.3
既往化疗次数		
<5次	19	46.3
≥5次	22	53.7
既往造血干细胞移植情况		
自体	1	2.4
异基因	5	12.2
无	35	85.4
CAR-T细胞来源		
自体	37	90.2
异基因供者	4	9.8
既往是否应用TKI治疗		
是	13	31.7
否	28	68.3
输注前髓外病变		
无	32	78.1
中枢神经系统侵犯	3	7.3
睾丸侵犯	1	2.4
骨骼和（或）软组织侵犯	5	12.2
细胞遗传学及分子学异常		
正常	9	22.0
未检测	4	9.7
Ph^+^伴T315I突变	10	24.4
Ph^+^不伴T315I突变	3	7.3
MLL-AF4阳性	1	2.4
IKZF1缺失突变	1	2.4
染色体超二倍体	2	4.9
染色体复杂核型	2	4.9
其他	9	22.0

注：CAR-T细胞：嵌合抗原受体T细胞；TKI：酪氨酸激酶抑制剂

2. 疗效：在中位第15（9～47）天，41例患者完全缓解和伴血液学不完全恢复的完全缓解（CR/CRi）率为95.1％（39/41），其中38例患者骨髓MRD阴性；2例患者对治疗无反应。2例患者于鼠源CD19 CAR-T细胞治疗后无应答后1个月左右接受hCART19s治疗，均达CR。1例患者在应用托珠单抗和糖皮质激素治疗CRS后出现CAR-T细胞扩增不良，首次评估未缓解，1个月后第二次输注hCART19s后获得CR。41例患者1年OS率为73.6％（95％*CI* 55.2％～92.3％），1年PFS率为56.2％（95％*CI* 38.1％～75.2％），中位OS、PFS均未达到。17例CR/CRi未接受进一步治疗，70.6％（12/17）的患者在随访结束时仍处于缓解状态，输注最早的两例患者PFS达12.6个月；其余5例患者在中位94（53～106）d出现疾病进展，包括3例因复发死亡，1例未复发但因消化道出血死亡，1例复发后带病生存。另外17例CR/CRi患者缓解后行巩固性造血干细胞移植（10例）或CD22 CAR-T细胞序贯治疗（7例），76.5％（13/17）的患者在随访结束时仍处于缓解状态；其余4例患者在中位第154（41～297）天出现疾病进展，包括1例移植后复发死亡，1例CD22 CAR-T细胞治疗后复发死亡及2例移植后复发带病生存。其余5例CR/CRi患者未接受巩固性治疗，于CAR-T细胞治疗后中位第72（55～115）天复发，均为CD19阴性复发。复发后3例患者接受了挽救性造血干细胞移植，均达骨髓MRD阴性的CR，1例持续缓解状态，其余2例分别于移植后2、3个月再次复发。2例患者行CD22 CAR-T细胞诱导治疗，1例达MRD阴性的CR，1个月后复发；另1例达骨髓MRD阳性的CR，桥接异基因造血干细胞移植后达MRD阴性且持续缓解。CAR-T细胞来源于移植供者的4例患者在CAR-T细胞治疗后均获得持续缓解，除1例再次输注CD19 CAR-T细胞行巩固性治疗，其余3例患者均未再次接受其他治疗。截至随访结束，共7例患者死亡，其中4例在CAR-T细胞输注后未接受进一步治疗，1例接受巩固性移植后复发，1例接受巩固性CD22 CAR-T细胞治疗后复发，1例CAR-T细胞输注后未缓解。1例患者死于弥漫性肺泡出血，该患者发生4级血小板减少（PLT 2×10^9^/L），凝血功能轻度异常（APTT正常，PT延长2 s，纤维蛋白原1.78 g/L）；另1例患者死于消化道出血，该患者同样出现4级血小板减少（PLT 23×10^9^/L），凝血功能异常（APTT延长23 s，PT延长4 s，纤维蛋白原0.56 g/L）；其余5例患者均死于疾病进展。

3. 不良反应：本研究中41例患者均发生了CRS，63.4％（26/41）为1～2级，36.6％（15/41）为3级，无1例患者发生4～5级CRS，症状可控。CAR-T细胞输注后定期检测患者外周血指标（铁蛋白、CRP、LDH、IL-2、IL-4、IL-6、IL-10、IFN-γ、IL-17a），相关性分析发现CRS等级与外周血IFN-γ峰值、IL-6峰值、CRP峰值呈正相关（*r*分别为0.359、0.349、0.415）。3～5级CRS患者外周血IFN-γ峰值、IL-6峰值高于0～2级CRS患者（*P*＝0.008，*P*＝0.047）（图1）。虽然有68％（28/41）的患者铁蛋白>500 µg/L，63％（26/41）的患者铁蛋白>1000 µg/L，但铁蛋白水平与CRS等级无相关性。3例患者发生免疫效应细胞相关神经毒性综合征（ICANS），分别为2、3、4级，其中2例既往有中枢神经系统白血病浸润。所有患者治疗期间均发生3～4级血液学不良反应，4级粒细胞减少中位持续时间为7（1～47）d，4级血小板减少中位持续时间为4（1～84）d，4级贫血减少中位持续时间为4（1～66）d。21例患者发生肝功能异常（谷丙转氨酶升高），7例患者发生肾功能异常（血肌酐升高），经对症支持治疗均恢复正常。1例输注供者来源的hCART19s细胞的患者发生了Ⅰ度（改良Glucksberg分级标准）急性移植物抗宿主病（aGVHD），表现为输注后16 d逐渐出现四肢斑丘疹，同时伴眼干、眼部分泌物增加，予糖皮质激素治疗后好转。

**图1:**
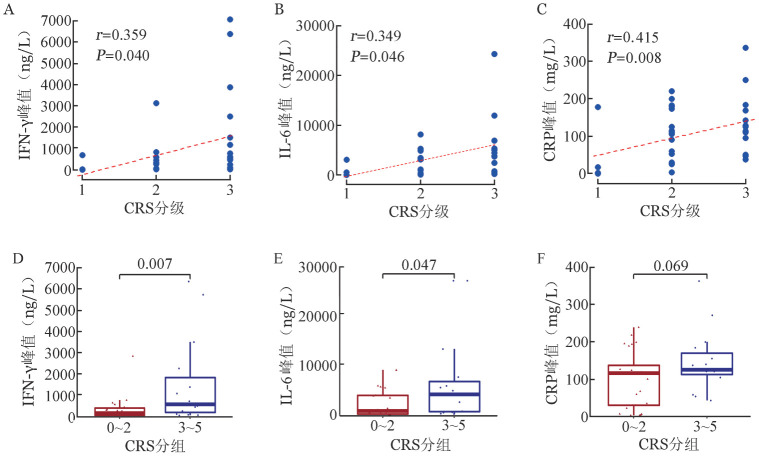
细胞因子释放综合征（CRS）等级与外周血IFN-γ峰值（A）、IL-6峰值（B）、C反应蛋白（CRP）峰值（C）的相关性及0～2级与3～5级CRS患者外周血IFN-γ峰值（D）、IL-6峰值（E）、CRP峰值（F）水平比较

## 讨论

CD19 CAR-T细胞疗法自应用于临床以来，已经在复发/难治B-ALL中展现出了良好的疗效，CR率可达70％～90％[5]–[7]。本研究中41例患者的CR/CRi率为95.1％，其中38例骨髓MRD阴性，优于既往的研究结果。值得注意的是，本研究中2例鼠源CD19 CAR-T细胞治疗后无应答的患者，在本次hCART19s细胞输注后均达CR。Cao等[11]的研究也表明，既往输注鼠源CD19 CAR-T细胞缓解后复发的患者，仍能受益于hCART19s细胞。这表明hCART19s细胞可以作为鼠源CD19 CAR-T细胞治疗无效或缓解后复发的有效治疗手段。一项系统综述显示，CD19 CAR-T细胞治疗难治复发B-ALL的平均1年OS率为58％，平均1年PFS率为37％[7]。本研究中41例患者1年OS率为71.9％（95％*CI* 55％～95％），1年PFS率为54.1％（95％*CI* 38％～78％）。表明应用hCART19s细胞治疗复发/难治B-ALL不仅具有较高的缓解率，还能改善生存。

CRS是CAR-T细胞最常见的不良反应，是由全身免疫细胞如单核细胞和巨噬细胞等的过度激活和增殖引起的全身炎症反应综合征。在我们的研究中，所有患者均出现了CRS症状和体征，严重CRS（≥3级）的发生率为36.6％，略高于既往研究的平均发生率[12]，可能与本研究中68.3％的患者输注前骨髓肿瘤负荷≥20％有关。本研究中大部分患者CRS主要表现为发热、乏力，经糖皮质激素、托珠单抗治疗后症状均可缓解。但值得关注的是，本研究中有1例患者在应用托珠单抗和糖皮质激素治疗后出现CAR-T细胞扩增不良，首次评估未缓解。我们在1个月后再次对该患者输注hCART19s细胞，患者在第二次输注后获得CR。多项临床研究表明，早期使用常规剂量的糖皮质激素和（或）托珠单抗治疗CRS不会对临床疗效造成影响[13]–[15]，但长时间或大剂量使用糖皮质激素可能会抑制患者体内CAR-T细胞的扩增和持久性[16]，累积使用糖皮质激素剂量过高的患者具有更短的PFS和OS期[17]。因此，使用过高剂量的糖皮质激素，可能是该患者第1次hCART19s输注无效的原因。ICANS是CAR-T细胞疗法另一个常见的不良反应，其发病机制暂不明确，有研究表明可能与内皮细胞活化和血脑屏障破坏有关[18]。本研究中2例发生了ICANS的患者既往有中枢神经系统白血病浸润，可能存在潜在的血脑屏障的损害，从而导致ICANS的发生。此外，异基因CAR-T细胞输注后出现aGVHD的情况在既往的几项研究中[19]–[21]均有报告，主要累及肠道、肝脏、皮肤，大多数患者通过糖皮质激素、环孢素A等治疗后症状可控。本研究中有1例患者输注了供者来源的CAR-T细胞后发生了aGVHD，累及皮肤和眼部，症状较轻，经糖皮质激素治疗后好转。

尽管CD19 CAR-T细胞治疗具有较高的CR率，但仍有30％～50％的患者在治疗后1年内复发[22]–[23]。复发的原因主要是CD19抗原表位的丢失[24]–[25]和CAR-T细胞在体内持续的时间有限[26]。本研究中5例CR患者未接受巩固性治疗，在CAR-T细胞治疗后中位第72（55～115）天复发，均为CD19阴性复发。说明虽然人源化抗体修饰通过降低免疫原性来延长CAR-T细胞的持久性，可以在一定程度上提高治疗效果，但复发仍旧难以避免。本研究中17例患者缓解后行巩固性造血干细胞移植（10例）或CD22 CAR-T序贯治疗（7例），76.5％（13/17）的患者在随访结束时仍处于缓解状态。Hu等[27]的研究表明，CD19 CAR-T细胞治疗后达CR的患者桥接HSCT可有效降低复发率。Pan等[28]的研究表明序贯输注CD19和CD22 CAR-T细胞治疗复发/难治B-ALL可延长缓解持续时间，其机制可能是防止抗原逃逸和延长CART细胞在体内存在的持续时间。因此，我们建议首次输注hCART19s治疗后达CR的患者尽早桥接造血干细胞移植或序贯CD22 CAR-T细胞治疗以期达到长期缓解。但因样本量有限，本研究并未对hCART19s治疗后达CR的患者接受巩固性移植和序贯CD22 CAR-T细胞治疗的疗效进行比较。CAR-T细胞输注后达CR的患者选择何种方案进行巩固性治疗更优，需要在更大的样本量、更长随访时间的前瞻性临床试验中加以探索。另外值得注意的是，在本研究中CAR-T细胞来源为供者的4例患者，在CAR-T细胞治疗后均获得持续缓解。马润芝等[21]报告了9例造血干细胞移植后复发的患者接受供者CD19 CAR-T细胞治疗的疗效，CR率为100％，44％（4/9）的患者无病生存达2年以上，其中2例无白血病生存期超过35个月。说明使用供者来源的CAR-T细胞或许可以作为减少复发的又一策略。

总之，hCART19s治疗复发/难治B-ALL患者具有较好的近期疗效，安全性可控。但由于本研究样本量有限，随访时间较短，长期疗效有待扩大样本量延长随访时间、进一步探索。

## References

[1] Bhojwani D, Pui CH (2013). Relapsed childhood acute lymphoblastic leukaemia[J]. Lancet Oncol.

[2] Crotta A, Zhang J, Keir C (2018). Survival after stem-cell transplant in pediatric and young-adult patients with relapsed and refractory B-cell acute lymphoblastic leukemia[J]. Curr Med Res Opin.

[3] Curran KJ, Margossian SP, Kernan NA (2019). Toxicity and response after CD19-specific CAR T-cell therapy in pediatric/young adult relapsed/refractory B-ALL[J]. Blood.

[4] Locatelli F, Zugmaier G, Rizzari C (2021). Effect of Blinatumomab vs Chemotherapy on Event-Free Survival Among Children With High-risk First-Relapse B-Cell Acute Lymphoblastic Leukemia: A Randomized Clinical Trial[J]. JAMA.

[5] Pan J, Yang JF, Deng BP (2017). High efficacy and safety of low-dose CD19-directed CAR-T cell therapy in 51 refractory or relapsed B acute lymphoblastic leukemia patients[J]. Leukemia.

[6] Zhang X, Lu XA, Yang J (2020). Efficacy and safety of anti-CD19 CAR T-cell therapy in 110 patients with B-cell acute lymphoblastic leukemia with high-risk features[J]. Blood Adv.

[7] Anagnostou T, Riaz IB, Hashmi SK (2020). Anti-CD19 chimeric antigen receptor T-cell therapy in acute lymphocytic leukaemia: a systematic review and meta-analysis[J]. Lancet Haematol.

[8] Lamers CH, Willemsen R, van Elzakker P (2011). Immune responses to transgene and retroviral vector in patients treated with ex vivo-engineered T cells[J]. Blood.

[9] Dwivedi A, Karulkar A, Ghosh S (2021). Robust Antitumor Activity and Low Cytokine Production by Novel Humanized Anti-CD19 CAR T Cells[J]. Mol Cancer Ther.

[10] Ma F, Ho JY, Du H (2019). Evidence of long-lasting anti-CD19 activity of engrafted CD19 chimeric antigen receptor-modified T cells in a phase I study targeting pediatrics with acute lymphoblastic leukemia[J]. Hematol Oncol.

[11] Cao J, Wang G, Cheng H (2018). Potent anti-leukemia activities of humanized CD19-targeted Chimeric antigen receptor T (CAR-T) cells in patients with relapsed/refractory acute lymphoblastic leukemia[J]. Am J Hematol.

[12] Lei W, Xie M, Jiang Q (2021). Treatment-Related Adverse Events of Chimeric Antigen Receptor T-Cell (CAR T) in Clinical Trials: A Systematic Review and Meta-Analysis[J]. Cancers (Basel).

[13] Liu S, Deng B, Yin Z (2020). Corticosteroids do not influence the efficacy and kinetics of CAR-T cells for B-cell acute lymphoblastic leukemia[J]. Blood Cancer J.

[14] Locke FL, Ghobadi A, Jacobson CA (2019). Long-term safety and activity of axicabtagene ciloleucel in refractory large B-cell lymphoma (ZUMA-1): a single-arm, multicentre, phase 1-2 trial[J]. Lancet Oncol.

[15] Topp MS, van Meerten T, Houot R (2021). Earlier corticosteroid use for adverse event management in patients receiving axicabtagene ciloleucel for large B-cell lymphoma[J]. Br J Haematol.

[16] Davila ML, Riviere I, Wang X (2014). Efficacy and toxicity management of 19-28z CAR T cell therapy in B cell acute lymphoblastic leukemia[J]. Sci Transl Med.

[17] Strati P, Ahmed S, Furqan F (2021). Prognostic impact of corticosteroids on efficacy of chimeric antigen receptor T-cell therapy in large B-cell lymphoma[J]. Blood.

[18] Gust J, Hay KA, Hanafi LA (2017). Endothelial Activation and Blood-Brain Barrier Disruption in Neurotoxicity after Adoptive Immunotherapy with CD19 CAR-T Cells[J]. Cancer Discov.

[19] Hu Y, Wang J, Wei G (2019). A retrospective comparison of allogenic and autologous chimeric antigen receptor T cell therapy targeting CD19 in patients with relapsed/refractory acute lymphoblastic leukemia[J]. Bone Marrow Transplant.

[20] Chen Y, Cheng Y, Suo P (2017). Donor-derived CD19-targeted T cell infusion induces minimal residual disease-negative remission in relapsed B-cell acute lymphoblastic leukaemia with no response to donor lymphocyte infusions after haploidentical haematopoietic stem cell transplantation[J]. Br J Haematol.

[21] 马 润芝, 何 祎, 杨 栋林 (2021). 供者CD19 CAR-T细胞治疗急性B淋巴细胞白血病移植后复发九例临床观察[J]. 中华血液学杂志.

[22] Maude SL, Laetsch TW, Buechner J (2018). Tisagenlecleucel in Children and Young Adults with B-Cell Lymphoblastic Leukemia[J]. N Engl J Med.

[23] Park JH, Rivière I, Gonen M (2018). Long-Term Follow-up of CD19 CAR Therapy in Acute Lymphoblastic Leukemia[J]. N Engl J Med.

[24] Orlando EJ, Han X, Tribouley C (2018). Genetic mechanisms of target antigen loss in CAR19 therapy of acute lymphoblastic leukemia[J]. Nat Med.

[25] Gardner R, Wu D, Cherian S (2016). Acquisition of a CD19-negative myeloid phenotype allows immune escape of MLL-rearranged B-ALL from CD19 CAR-T-cell therapy[J]. Blood.

[26] Mueller KT, Maude SL, Porter DL (2017). Cellular kinetics of CTL019 in relapsed/refractory B-cell acute lymphoblastic leukemia and chronic lymphocytic leukemia[J]. Blood.

[27] Hu L, Charwudzi A, Li Q (2021). Anti-CD19 CAR-T cell therapy bridge to HSCT decreases the relapse rate and improves the long-term survival of R/R B-ALL patients: a systematic review and meta-analysis[J]. Ann Hematol.

[28] Pan J, Zuo S, Deng B (2020). Sequential CD19-22 CAR T therapy induces sustained remission in children with r/r B-ALL[J]. Blood.

